# Microstructure-informed constitutive modeling of granular media under multidirectional loading: From particle-scale to continuum

**DOI:** 10.1038/s44172-026-00652-1

**Published:** 2026-04-30

**Authors:** Nazanin Irani, Pegah Golestaneh, Mohammad Salimi, Merita Tafili, Yuha Park, Johannes Lederer

**Affiliations:** 1 Chair of Soil Mechanics, Foundation Engineering, and Environmental Geotechnics, Ruhr-University Bochum, Bochum, Germany; 2 Chair of Data-Driven Mathematics, University of Hamburg, Hamburg, Germany

**Keywords:** Civil engineering, Applied physics, Computational science

## Abstract

Simulating the response of granular materials under realistic loading scenarios is essential for ensuring the reliability of geotechnical infrastructure. This task is particularly challenging because natural soils exhibit inherently non-uniform particle arrangements due to gravitational sedimentation and are subjected to complex, multidirectional loading conditions from environmental forces such as wind and seismic activity. Unlike crystalline solids, there is no closed-form mathematical framework that fully describes soil’s collective response. In engineering practice, this complexity is typically addressed using nonlinear constitutive models calibrated against laboratory data. However, such data are often specific to the site and material, influenced by variations in soil type, particle morphology, experimental apparatus, and loading conditions, making them difficult to generalize. The discrete element method (DEM) offers a unique pathway to overcome these limitations by providing direct access to particle-scale kinematics, contact forces, and evolving microstructure. As assemblies of particles exhibit chaotic rearrangements under loading, predicting their collective behavior becomes highly nonlinear and computationally intensive. Here, deep-learning models offer a promising route to replicate these complex relationships. In this work, we develop a deep-learning model using DEM simulations to address fundamental challenges in predicting the response of granular media under multidirectional loading paths, with direct applications to pressing engineering problems such as optimizing wind turbine foundations.

## Abstract (web summary)

Nazanin Irani and colleagues introduce a novel microstructure-informed deep-learning framework, trained on discrete element simulations, to predict the stress response of granular soils under multidirectional loading. The model enables estimation of soil stress response from a known initial state, particle aspect ratio, and gravitationally induced particle arrangement under prescribed strain paths.

## Introduction

Granular materials, such as sands, tailings, and ballast, exhibit inherently complex mechanical behavior arising from their discrete nature, irregular particle shapes, and highly variable initial arrangements^1–5^. Unlike crystalline solids, no closed-form mathematical framework exists to describe their collective response, which is strongly influenced by evolving microstructural mechanisms and chaotic particle rearrangements during loading^6,7^.

**Fig. 1:**
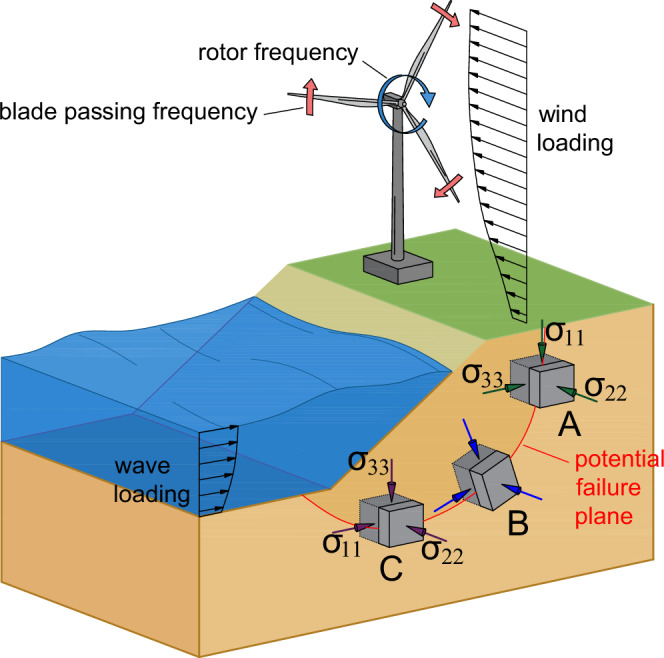
A typical example of multidirectional loading condition. Wind turbine foundation subjected to multidirectional loading from rotor frequency, blade passing frequency, and wind forces, while adjacent slope experiences rotation of principal stress directions at representative elements A, B, and C. Each element shows distinct orientations of principal stresses (*σ*_11_, *σ*_22_ and *σ*_33_).

Simulating soil behavior is crucial in geotechnical engineering, particularly in the design and long-term performance assessment of critical infrastructures^8^. Energy transition systems–such as wind turbine foundations–and tailings storage facilities must withstand complex, multidirectional loading conditions generated by wind, waves, tides, ocean currents, and seismic events^9–11^. An example of such loading conditions is shown in Fig. 1, which depicts a realistic scenario of loads acting on the foundation of a wind turbine installed on sloped terrain. The scalars *σ*_11_, *σ*_22_, and *σ*_33_ represent the major, minor, and intermediate effective principal stresses, respectively, applied to representative subsurface elements such as points *A*, *B*, or *C*. In a simple slope, spatial variations in the orientation of principal stresses produce zones of compression, shear, and extension. Near the crest, the major principal stress (*σ*_11_) is oriented nearly vertically, indicating compression; mid-slope, the principal stresses rotate along the potential failure plane, driving active shear deformation; near the toe, *σ*_11_ becomes nearly horizontal, reflecting extension^12^. Civil engineering structures situated on such slopes inevitably experience time-varying combinations of vertical, horizontal, and rotational forces^13^, making accurate simulation of granular material behavior under these conditions both essential and challenging.

Three core factors underpin this challenge. First, most constitutive models are developed and validated using axisymmetric laboratory tests (*σ*_22_ = *σ*_33_ ≠ *σ*_11_) or simplified stress paths where one principal stress is fixed, affecting their applicability to complex loading. Second, information on the evolution of microstructural quantities under realistic loading paths remains limited. A key example is soil fabric, defined as the spatial arrangement and orientation of particles and voids. Tracking fabric evolutions requires advanced imaging techniques–such as X-ray computed tomography, photoelasticity, or stereophotogrammetry–which are costly and not widely accessible in many soil laboratories^14–16^. Third, advanced constitutive models often include more than twelve material parameters. Calibrating these models may require high-quality experimental data, a process that is both time-consuming and sensitive to variability. To overcome these limitations, physics-based particle simulations, such as the DEM, offer a unique advantage by directly resolving particle-scale interactions and fabric evolution, thereby providing micro-to-macro insights that are difficult or impossible to obtain experimentally^17,18^. However, as microstructure of particle assemblies evolve chaotically under complex loading, directly predicting their response remains computationally demanding. Deep learning provides a complementary solution: as universal function approximators^19,20^, neural networks can learn intricate, nonlinear relationships from DEM-generated datasets, bypassing the need for manually defined constitutive equations and extensive parameter calibration^21^. Recent deep-learning applications in granular mechanics^22–25^ have shown great promise. However, most existing studies have been validated solely under conventional triaxial loading conditions, without accounting for critical particle-scale characteristics such as particle shape. As a result, the micro-to-macro response of granular materials under more general, multidirectional loading–such as true triaxial conditions–remains largely unexplored. Moreover, deep learning is known to be “data-hungry”^26^, which makes extensive and high-quality DEM-data mandatory for training those methods.

In this study, we present a systematic micro-to-macro scale analysis of granular materials using an extensive set of DEM simulations, coupled with a novel deep-learning framework. The DEM dataset encompasses 260 true triaxial simulations conducted on granular assemblies featuring diverse particle shapes (spherical and elongated), initial fabric defined by bedding plane orientations of 0°, 45°, 90°, a spectrum of initial void ratios spanning loose to dense states, and varied stress conditions. From this, we train a deep-learning model that ingests both microstructural (fabric tensor, particle shape) and macrostate descriptors (void ratio, initial stress) and predicts the evolving stress response throughout loading. The proposed model is among the first to capture the response of soils with different particle shapes and fabrics under true triaxial conditions using a unified parameter set. Our approach reduces reliance on empirical calibration, improves generalizability, and provides a computationally efficient tool for element-level prediction of granular behavior in complex engineering applications.

## Methods

### DEM methodology

DEM is a particle-based computational technique widely used to investigate the mechanical response of granular materials by explicitly simulating the motion and interactions of individual grains^27^. Unlike continuum approaches, DEM treats each particle as an independent rigid body governed by Newton’s laws and contact mechanics, providing direct resolution of inter-particle forces and displacements. Granular assemblies are constructed by generating particles arranged to represent natural or engineered materials, enabling bottom-up insights into macroscopic behavior by capturing microscale mechanisms such as force chains, contact fabric, and deformation patterns. The theoretical background is detailed in Salimi et al.^7,18,28,29^, and a concise summary is provided here.

The macroscopic stress tensor of a granular assembly under applied boundary conditions can be expressed from the discrete contact forces and branch vectors as^30,31^:

1 $${\boldsymbol{\sigma }}=\frac{1}{V}\mathop{\sum }\limits_{c=1}^{{N}_{c}}{{\bf{l}}}^{c}\otimes {{\bf{f}}}^{c},$$

where *V* is the assembly volume, *N*_*c*_ the total number of contacts, **f**^*c*^ the contact force, and **l**^*c*^ the branch vector linking a particle center to its contact point *c*. The operator  ⊗ indicates the dyadic product between two vectors. The micro-to-macro stress formulation and the explicit use of contact force follow the granular micromechanics paradigm, in which continuum stresses emerge from force-branch statistics and anisotropic contact networks. Within granular micromechanics, elastic response may become tension-compression asymmetric (bimodulus) as a microstructural effect; such asymmetry can propagate to structural members (e.g., beams) and should be expected whenever force-chain topology differs in tension and compression^32,33^. From ***σ***, the mean effective stress *p* = *σ*_*ii*_/3, deviatoric stress $q=\sqrt{3{\sigma }_{ij}^{* }{\sigma }_{ij}^{* }/2}$, and Lode angle *θ* are calculated, providing a full description of the stress state and enabling controlled exploration of principal stress rotation effects. Here, ${\sigma }_{ij}^{* }={\sigma }_{ij}-{\delta }_{ij}p$ denotes the deviatoric component of the stress tensor, where *δ*_*ij*_ is the Kronecker delta. The Lode angle (*θ*) is then calculated as:

2 $$\cos (3\theta )=\frac{{J}_{3}}{2}{\left(\frac{3}{{J}_{2}}\right)}^{2},$$

with ${J}_{2}=| | {\sigma }_{ij}^{* }| |$ and ${J}_{3}=\det (\mathop{\sigma }\nolimits^{* }_{ij})$ the second and third invariants of the deviatoric stress tensor, respectively. $\det ({A}_{ij})$ indicates the determinant of the tensor ***A***. The invariant-based formulation adopted in Eq. (2) provides a coordinate-invariant and unambiguous characterization of stress paths under true triaxial loading. Variations in *θ* effectively reorient the dominant force chains and alter the contact topology, leading to a co-evolution of the inter-particle contact fabric tensor and the resulting anisotropy.

The microstructure of granular materials is characterized by the spatial distribution and orientation of particles, voids, and contacts, collectively termed the “fabric”^34–37^. Among these, the contact fabric tensor,

3 $${\bf{F}}=\frac{1}{2{N}_{c}}\mathop{\sum }\limits_{k=1}^{2{N}_{c}}{{\bf{n}}}^{k}\otimes {{\bf{n}}}^{k},$$

quantifies the directional distribution of unit contact normal vectors **n**^*k*^, reflecting the connectivity and anisotropy of the load-bearing skeleton^38,39^. DEM uniquely enables tracking the evolution of **F** concurrently with macroscopic stress-strain responses, providing critical micro-to-macro insights into deformation mechanisms. Treating fabric as an internal variable is coherent with micro-mechanically informed damage and strain-gradient frameworks, where energy storage/dissipation and intrinsic length scales stem from granular interactions. Such models provide well-posedness in the presence of evolving anisotropy and localization, and naturally accommodate microstructure-controlled softening^40,41^.

**Fig. 2:**
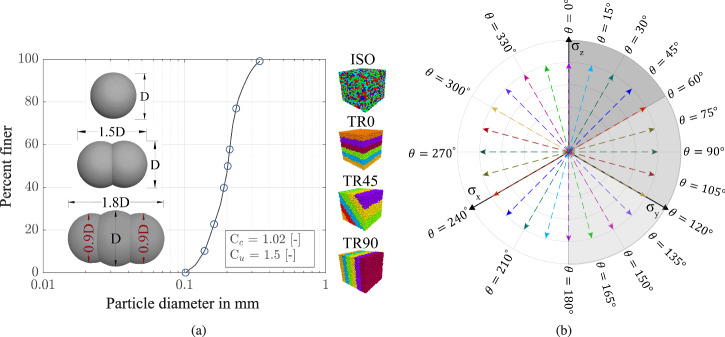
Characteristics of particulate assemblies and loading paths. **a** Particle size distribution and particle shapes used in simulations. **b** stress paths applied in DEM simulations at fixed Lode angles from *θ* = 0^∘^ to 180^∘^.

For sample preparation, a granular assembly was numerically generated to fill a cubic domain of 5 × 5 × 5 mm^3^, bounded by pairs of frictionless faceted walls subjected to prescribed stress conditions. To systematically investigate the influence of particle shape on the mechanical behavior of granular media, three aspect ratios (AR) were considered: spherical particles (AR = 1.0), elongated particles composed of two overlapping spheres (AR = 1.5), and elongated particles composed of three overlapping spheres (AR = 1.8). The diameter of the pebbles was determined using an optimization method proposed by Price et al.^42^. Figure 2 depicts the different particle shapes and presents the corresponding particle size distribution curve of the generated assemblies. The synthetic material mimics a poorly graded sand, characterized by a coefficient of uniformity *c*_*u*_ = 1.02 and a coefficient of curvature *c*_*c*_ = 1.5. We created various specimens with different initial fabrics, including isotropic specimens labeled as ‘ISO’ and three transversely isotropic ones labeled as ‘TR0’, ‘TR45’ & ‘TR90’ with bedding plane angles of 0°, 45°, and 90° (see Fig. 2).

In alignment with previous studies^43–48^, a linear contact model was employed to model particle interactions at their contact points. The DEM parameters utilized in this investigation are detailed in Table 1. Given that silica sand typically displays an effective Young’s modulus ranging from 10.60 to 12.70 MPa^49,50^, we adopted a reference stiffness of 10^5^ kPa for the sample under a mean effective stress of 100 kPa^45,51^. To model the behavior of natural sand, which typically has an internal friction angle of approximately 30°, we adopted an inter-particle friction coefficient *μ* of 0.5, as recommended in the literature^45,52^. To ensure quasi-static conditions during the simulations, a strain rate lower than 5 × 10^−5^ s^-1^ is generally advised^53^, with an inertial number kept below 0.001 s^-1^^54^. Accordingly, a strain rate of 0.005 s^-1^ was chosen, along with a time step of 1.5 × 10^−7^ s. A summary of the DEM parameters used in the simulations is provided in Table 1.

**Table 1:** DEM simulation parameters (with *k*_0_ = 10^8^ kN/m^2^)

Parameter	Value
Particle density, *ρ*_*p*_	2650 kg/m^3^
Inter-particle friction coefficient, *μ*	0.5
Damping ratio	0.7
Wall stiffness	10^7^ kN/m
Tangential contact stiffness, *k*_*s*_	*k*_0_ ⋅ *r*
Normal contact stiffness, *k*_*n*_	*k*_*s*_

The strain path in the z-direction is imposed using a servo-control mechanism that continuously computes and adjusts the strains in the x- and y-directions to maintain constant Lode angle values. Based on the stress states schematically illustrated in Fig. 2(b), each DEM simulation was conducted at a constant mean effective stress and a fixed Lode angle. This setup enables a systematic investigation into the influence of principal stress rotation on the mechanical response of granular media under distinct multidirectional loading paths. Lode angles ranging from *θ* = 0° to *θ* = 180°, in 15° increments, are considered to capture the full spectrum of multidirectional stress conditions. The simulated stress paths reflect key real-world geotechnical loading scenarios and are systematically characterized by the Lode angle. Specifically, *θ* = 0° corresponds to conventional triaxial compression, *θ* ≈ 30° represents near-plane strain conditions relevant to strip foundations, tunnels, slope stability analyses, and long retaining walls; and *θ* = 60° corresponds to conventional triaxial extension, as encountered in excavation-induced unloading, tunnel face instability, and stress relief around underground openings. Characterizing soil response over a broad range of Lode angles enables the capture of non-axisymmetric stress states representative of complex loading conditions such as seismic shearing and offshore wind turbine foundation responses. To further assess the influence of the initial stress state and void ratio, a series of loose, medium-dense, and dense assemblies were simulated and sheared under varying initial mean effective stresses, *p*_0_, ranging from 50 to 600 kPa. A summary of the DEM tests, which includes sample deposition types, particle shapes, initial mean effective stress values, initial void ratios and fabric tensor components for the simulated samples, is provided in Supplementary Table 1.

### Deep-learning methodology

In recent years, deep learning has increasingly adopted advanced architectures, including Long Short Term Memory (LSTMs) networks and attention-based models such as Transformers^55^. These models have proven highly effective in tasks involving sequential or temporal data—such as natural language processing, time series prediction, and the analysis of video and motion data—due to their ability to capture long-range dependencies. However, our task fundamentally differs from these applications. We focus on static input-output mappings, where each material state independently mapped to a corresponding stress response. This setting doesn’t require memory of past states or any form of temporal processing. Given these considerations, we employ a multi-layer feed-forward neural network, motivated by its ability to approximate complex static input-output relationships with high fidelity. This design decision is grounded in theoretical findings and empirical evidence, reflecting a careful balance between model expressiveness^56–58^ and computational efficiency^59–61^. We further enhance our feed-forward network structure with two key components: 1- using the EvoLved Sign Momentum (Lion) optimizer^59^, which combines the adaptive qualities of Adaptive Moment Estimation (ADAM)^62^ with the memory efficiency of sign-based updates to achieve faster convergence and 2- introducing a hybrid loss function.

To comprehensively represent the stress response, we incorporate critical variables including the initial stress tensor, void ratio, particle AR, and the fabric tensor. These inputs encapsulate the soil’s micro structural features, stress history, and anisotropic characteristics that strongly influence its mechanical response. To guide the selection of input features, we performed a series of data-driven evaluations—most notably employing a random forest model—to quantify the correlation between each candidate input feature and the target stress components. The results of these analyses informed the final feature set used for training the model, which consists of the following eleven variables:

- Particle AR ∈ {1, 1.5, 1.8} corresponding to three different particle shapes
- Strain components in the x-, y- and z- directions, $[{\varepsilon }_{x}^{i},{\varepsilon }_{y}^{i},{\varepsilon }_{z}^{i}]$
- Initial values of the inter-particle contact fabric tensor in three directions, $[{F}_{x}^{0},{F}_{y}^{0},{F}_{z}^{0}]$
- Initial void ratio, *e*_0_
- Initial stress condition in the x-, y-, and z- directions, $[{\sigma }_{x}^{0},{\sigma }_{y}^{0},{\sigma }_{z}^{0}]$

Since the Lode angle depends on the third invariant of the stress tensor, specifying the initial stress magnitudes in all three directions inherently captures the effects of the initial Lode angle in the input data. It is important to note that the network should learn the variations in the Lode angle directly from the patterns present in the training data. Finally, we standardized both the input and output variables to ensure numerical stability and reliable convergence of our pipeline. We adopt StandardScaler from the sklearn.preprocessing module, which standardizes the characteristics by removing the mean and scaling to the unit variance.

From a mathematical point of view, our goal is to learn a nonlinear function that describes the stress-strain relationship. This function has eleven input variables and the three-dimensional stress response. We approximate the target function using a neural network *f*_*Ω*_ parameterized by weights and biases collectively denoted as *Ω*:

4 $${f}_{{\mathbf{\Omega }}}:\,{{\mathbb{R}}}^{11}\to {{\mathbb{R}}}^{3},\,{{\bf{x}}}^{i}\mapsto {\widehat{{\boldsymbol{\sigma }}}}^{i}.$$

Hence, we aim to learn **Ω** from data. This is done by minimizing the discrepancy between the predicted stress vector ${\widehat{{\boldsymbol{\sigma }}}}^{i}$ and the corresponding actual values in our DEM simulations. Once trained, the neural network can predict the stress response of granular materials under varying loading paths.

**Fig. 3:**
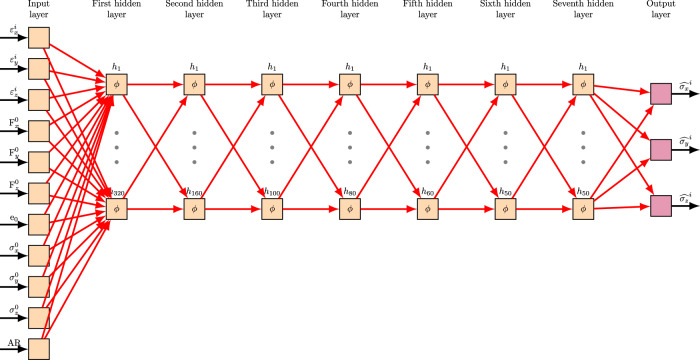
The proposed deep-learning model architecture. It consists of seven hidden layers with 320, 160, 100, 80, 60, 50, and 50 neurons, respectively. The input layer contains 11 neurons--- $AR,{\varepsilon }_{x}^{i},{\varepsilon }_{y}^{i},{\varepsilon }_{z}^{i},{F}_{x}^{0},{F}_{y}^{0},{F}_{z}^{0},{e}_{0},{\sigma }_{x}^{0},{\sigma }_{y}^{0},and\,{\sigma }_{z}^{0}$---while the output layer consists of three neurons, representing the predicted stress values denotes as ${\widehat{{\sigma }_{x}}}^{i},{\widehat{{\sigma }_{y}}}^{i},and\,{\widehat{{\sigma }_{z}}}^{i}$. We use the symbol “ ̂ ” for the network’s output.

Figure 3 illustrates the fully-connected network architecture. The activation function in hidden layers is the Leaky Rectified Linear Unit (Leaky ReLU) activation function, which is a variant of the popular ReLU function designed to avoid the dying-ReLU phenomenon^63^. It is defined as *ϕ*(*x*) = *x* if *x* ≥ 0 and *ϕ*(*x*) = *αx* if *x* < 0, where we choose *α* = 0.1 as the negative slope. The activation of the output layer is linear to allow for a full range of stress responses. Formally, at each time step *i* (rows in each data sample), the model receives an input vector

5 $${{\boldsymbol{x}}}^{i}=[{{\rm{AR}},\varepsilon }_{x}^{i},{\varepsilon }_{y}^{i},{\varepsilon }_{z}^{i},{F}_{x}^{0},{F}_{y}^{0},{F}_{z}^{0},{e}_{0},{\sigma }_{x}^{0},{\sigma }_{y}^{0},{\sigma }_{z}^{0}]^{\top },$$

consisting of the physical variables relevant to the stress output. This vector is passed through a sequence of fully connected layers. In each hidden layer (*l* ∈ {1, 2, 3, 4, 5, 6, 7}), the input is transformed according to

6 $${{\boldsymbol{H}}}^{(l)}=\phi ({{\boldsymbol{W}}}^{(l-1)}{{\boldsymbol{H}}}^{(l-1)}+{{\boldsymbol{b}}}^{(l)}),\,{{\boldsymbol{H}}}^{(0)}={{\boldsymbol{x}}}^{i}.$$

Here, ***W***^(*l*−1)^ is a weight matrix that maps between the consecutive layers with indexes *l* and (*l* − 1), and ***b***^(*l*)^ is the corresponding bias vector. The final output is computed via a linear projection of the last hidden layer:

7 $${\widehat{{\boldsymbol{\sigma }}}}^{i}={{\boldsymbol{W}}}^{(7)}{{\boldsymbol{H}}}^{(7)}.$$

This formulation represents the compact flow of information from raw input to produce the final predicted stress outputs. With the model architecture defined, we now turn to the training and optimization strategy.

The training strategy is to optimize the network parameters so that the discrepancy between the predicted and DEM stress components is minimized, while preserving the underlying stress patterns intrinsic to the data. To optimize network parameters, we used Lion optimizer, a state-of-the-art evolutionary approach to deep learning that combines the benefits of adaptive moment estimation with reduced memory requirements. Unlike traditional optimizers, Lion operates by tracking only the momentum term while maintaining an exponential moving average of gradients, leading to faster convergence and improved generalization^59^. The initial learning rate was set at 10^−4^, with a weight decay at 10^−2^ to ensure numerical stability. To further improve convergence, we used a learning rate scheduler using the ReduceLROnPlateau strategy. This scheduler monitors the validation loss and reduces the learning rate by a factor of 0.92 if no improvement is observed for four consecutive epochs, with a minimum change threshold of 10^−6^.

In addition to a suitable optimization algorithm, the selection of an appropriate loss function is crucial to guide the network to learn meaningful stress patterns. We employed smooth-*ℓ*_1_ loss as our “base” loss function, due to its balanced characteristics between Mean Squared Error (MSE) and Mean Absolute Error (MAE)^64^. Smooth-*ℓ*_1_ loss is particularly suitable for our regression problem, as it penalizes small errors quadratically, like MSE while treating larger errors linearly, like MAE. This hybrid behavior offers a practical compromise, maintaining sensitivity to the overall deviation while reducing sensitivity to outliers and extreme discrepancies. To better capture key physical transitions, we augment this loss function with a weighted scheme that emphasizes critical points that mark the onset of dynamic behavior. Given the varying length of the data samples (data points in each data sample), a proportional weighting strategy was adopted. Based on our knowledge of DEM, we introduced a weight factor of *λ* = 0.95 for the early part (seven-eleventh) and a complementary weight of (1 − *λ*) for the remaining part of each data sample. The resulting loss function is defined as

8 $${{\mathcal{L}}}_{\rm{custom}} = 	 \, \lambda \times \frac{{{\sum }_{i=1}^{{N}_{p}}{{\mathcal{L}}}_{\rm{smooth}}}-{\ell }_{1}({{\bf{y}}}^{i},{\widehat{{\bf{y}}}^{i})}}{{N}_{p}}+(1-\lambda ) \\ 	 \times \frac{{\sum }_{j={N}_{p}+1}^{N}{{\mathcal{L}}}_{{\rm{smooth}}-{\ell }_{1}}({{\bf{y}}}^{j},{\widehat{{\bf{y}}}}^{j})}{{N}_{r}},$$

where ${{\mathcal{L}}}_{smooth-{\ell }_{1}}$ is the notation for smooth-*ℓ*_1_ loss, *N* is the total number of data points in each data sample, *N*_*p*_ is denoted as the number of data points in the early part, and *N*_*r*_ = *N* − *N*_*p*_ counts the remaining data points. Here, ***y*** and $\widehat{{\boldsymbol{y}}}$ represent the actual value of our DEM simulations and the predicted values, respectively. More specifically, this custom loss function is designed to reflect the key mechanical transitions that occur in the small-strain regime, including initial strain hardening, subsequent softening, the attainment of peak stress, and the phase transformation point. Capturing these responses with high accuracy is essential, as they govern the evolution of stiffness, anisotropy, and dilatancy. At larger strains, the material typically approaches a steady-state condition, where continued deformation occurs with minimal variation in stress. In this regime, the stress response tends to follow more consistent patterns, making it relatively easier for the data-driven model to learn. Consequently, the model places greater emphasis on fidelity in small strains, where accurate prediction is more challenging yet crucial for capturing the fundamental aspects of soil behavior. In addition to our custom loss, which prioritizes prediction accuracy in the critical regions (e.g., those early data points), we further improve our pipeline with Probably Approximately Correct PAC-Bayes robust loss^65^. PAC-Bayes robust loss introduces a perturbation-based approach that evaluates worst-case scenarios, making the model more resilient to input variations and noise.

This approach, grounded in the PAC-Bayesian theory, has been shown to improve generalization under uncertainty. Motivated by this framework, we construct the final training objective as

9 $${{\mathcal{L}}}_{\rm{total}}={{\mathcal{L}}}_{\rm{custom}}+\gamma \times \mathop{\max }\limits_{s\in [1,S]}\left(\frac{1}{N}\mathop{\sum }\limits_{i=1}^{N}{{\mathcal{L}}}_{\rm{smooth}}-{\ell }_{1}({{\bf{y}}}^{i},{\widehat{{\bf{y}}}}^{i}+{{\boldsymbol{\xi }}}_{i}^{(s)})\right),$$

where *γ* is a robust weight, *S* is the number of perturbation samples, and ${{\boldsymbol{\xi }}}_{i}^{(s)}$ denotes perturbation noise drawn from Unif([−*ν*, *ν*]^3^). We set *S* = 3 and *γ* = 0.1. By combining this term with ${{\mathcal{L}}}_{\rm{custom}}$, the model assigns greater weight to outlier samples, and learns to remain stable under perturbations, leading to improved robustness and generalization.

## Results and discussion

### DEM simulations

This section presents results from a comprehensive series of DEM simulations designed to isolate the key micro- and macro-scale factors influencing the shear strength and volumetric response of granular materials. The simulations systematically investigate the effects of initial confining pressure (*p*_0_), initial void ratio (*e*_0_), Lode angle (*θ*), initial fabric, and particle AR. While the significance of *p*_0_ and *e*_0_ in constitutive modeling is well established, our findings reveal that neglecting the influence of *θ*, initial fabric, and particle AR can lead to substantial under- or overestimation of shear strength under complex loading paths. Overall, 260 DEM simulations were performed, although only a representative subset is presented here for clarity. In all cases, a positive volumetric strain denotes dilation, while a negative value indicates contraction. The effects of micro- and macro-scale parameters in shaping the stress-strain response are elaborated below.

**Fig. 4:**
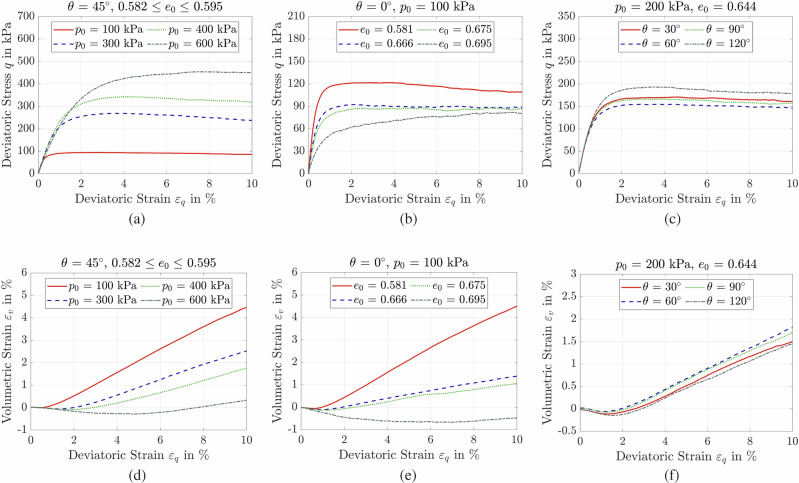
Influence of initial state and loading path on granular assembly response. Effects of **a**, **d** initial confining pressure *p*_0_, **b**, **e** initial void ratio *e*_0_, and **c**, **f** Lode angle *θ*.

Figure 4 (a & d) show the influence of *p*_0_ on the drained mechanical response of granular assemblies, with identical initial void ratio *e*_0_ and Lode angle *θ*. Increasing *p*_0_ from 100 to 400 kPa results in a noticeably stiffer stress-strain response, with higher maximum deviatoric stress reached at lower strain levels. This behavior reflects the enhanced inter-particle contact forces under greater confinement, which resist shear-induced rearrangement^21^. Correspondingly, volumetric strain curves indicate a marked reduction in dilative behavior at higher *p*_0_. The trends align well with experimental drained triaxial data^66–69^, lending confidence to the DEM predictions.

Figure 4b, e demonstrate the influence of *e*_0_ on the mechanical response of granular assemblies, all subjected to identical initial confining pressures and Lode angles. Looser assemblies (say higher *e*_0_) exhibit a more pronounced contractive volumetric response, stemming from greater freedom for particle rearrangement. In contrast, denser packings deform with reduced volumetric contraction. This trend also manifests in the stress-strain response: denser assemblies sustain higher maximum deviatoric stresses, while looser ones mobilize less shear resistance. These results agree with experimental observations^70–73^, confirming the ability of DEM model to capture the void ratio effects.

Figure 4c, f illustrate the role of *θ* in shaping the drained response of granular assemblies under identical *p*_0_ and *e*_0_. As a measure of the intermediate principal stress, *θ* governs the stress triaxiality and mediates transitions between compression- and extension-dominated regimes. The DEM results show a non-monotonic influence: increasing *θ* from 30° to 60° reduces maximum deviatoric stress while promoting dilation, whereas increasing *θ* from 90° to 120° yields higher *q* and more contractive behavior. This asymmetry reflects the complex, path-dependent interplay between stress anisotropy and evolving internal fabric. The results confirm that neglecting Lode angle effects can lead either to design vulnerability through overestimation of shear strength and deformation or to uneconomical outcomes due to underestimation of soil shear strength. From a modeling standpoint, most constitutive frameworks introduce Lode angle dependence through the third invariant of the stress tensor, yet their calibration often relies on limited datasets (typically *θ* = 0° or 90°). The present DEM dataset spans a broader *θ* range, offering valuable input for refining constitutive laws to capture non-axisymmetric loading effects.

The variations observed across *p*_0_, *e*_0_, and *θ* highlight that proportional changes in macro-scale entities effectively alter the degrees of freedom available to each granular element. This modulation of constraint–by varying confinement, density, and stress path–fundamentally governs the accessible kinematic rearrangements at the microscale, and hence the macroscopic shear and volumetric behavior. In this sense, stress path control is not merely a loading specification, but a means of steering the internal mechanics of the assembly.

**Fig. 5:**
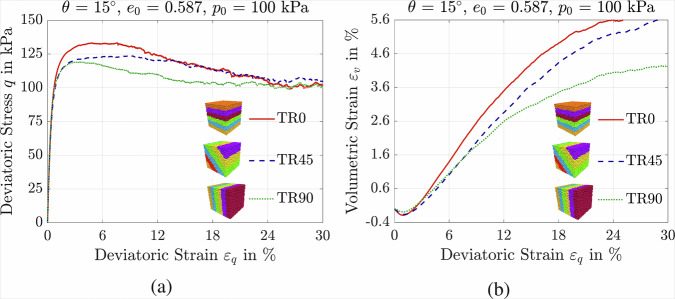
Influence of initial fabric on granular assembly response. Response of assemblies with bedding planes oriented at 0^∘^ (TR0), 45^∘^ (TR45), and 90^∘^ (TR90), under *p*_0_ = 100, *e*_0_ = 0.589, and *θ* = 15^∘^: **a** deviatoric stress-deviatoric strain plane; **b** volumetric strain-deviatoric strain plane.

Figure 5 illustrates the mechanical response of granular assemblies with an initial void ratio of *e*_0_ = 0.586, subjected to a confining pressure of *p*_0_ = 100 kPa and sheared at Lode angle of *θ* = 15°, considering different bedding plane orientations. A schematic representation of the bedding configurations is also provided in Fig. 5, where TR0 sample has a bedding plane angle of 0°, TR45 corresponds to 45°, and TR90 represents a vertical bedding plane at 90°. The results shown in Fig. 5a demonstrate that the deviatoric stress response is influenced by the orientation of the bedding plane. Specifically, increasing the bedding angle from 0° to 90° leads to a reduction in the maximum deviatoric stress from 136 kPa to 121 kPa. Neglecting the effects of initial fabric may therefore lead to an overestimation of soil shear strength and an unsafe design of geotechnical structures. Furthermore, as shown in Fig. 5b, samples with horizontal bedding planes exhibit a more dilative response compared to those with vertical bedding planes. Overall, the DEM results highlight that the initial stress state, loading path, and void ratio are fundamental factors influencing the shear strength and deformation behavior of particulate assemblies under loading. During the loading process, the internal arrangement of grains evolves depending on the direction and magnitude of the applied load, potentially leading to fabric anisotropy^17,29,74^. This anisotropy significantly influences soil behavior and, consequently, the performance of nearby geostructures^75^.

**Fig. 6:**
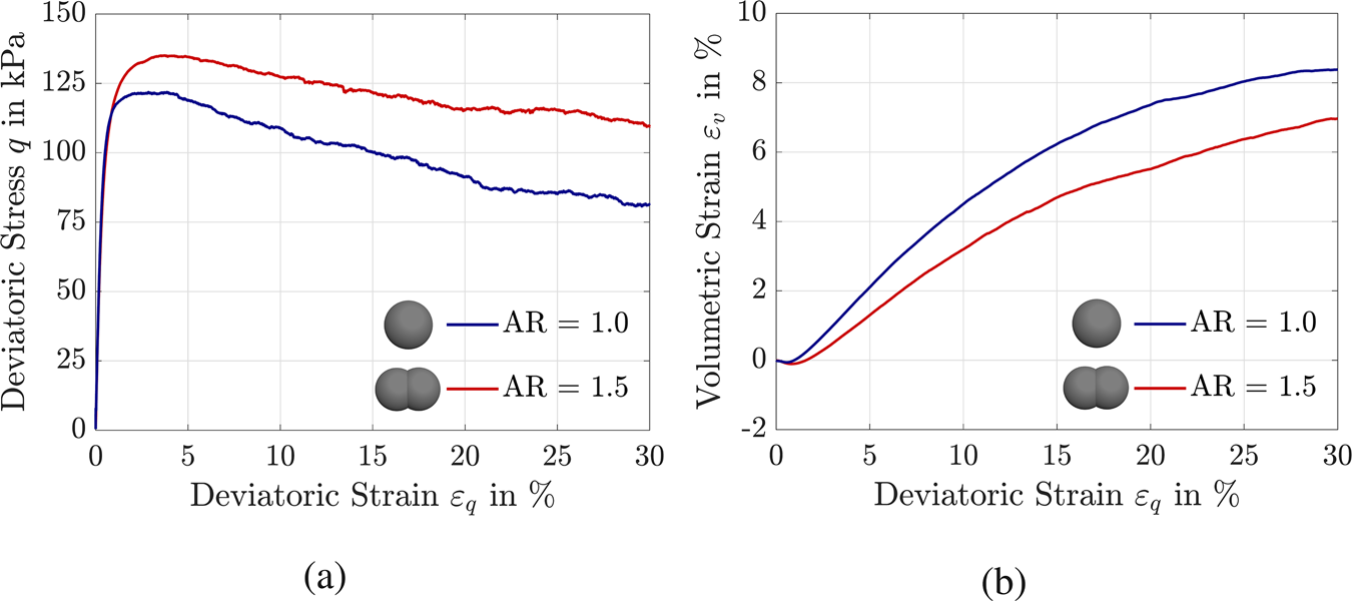
Effect of particle AR on ISO assembly response. Assemblies with *p*_0_ = 100, *D*_r_ = 42%, and *θ* = 0^∘^: **a** deviatoric stress-deviatoric strain plane; **b** volumetric strain-deviatoric strain plane.

Figure 6 illustrates the influence of particle AR on the stress-strain response of medium-dense ISO assemblies subjected to drained shearing with a Lode angle of *θ* = 0° and an initial mean effective stress of *p*_0_ = 100 kPa. In this series of tests, assemblies composed of particles with AR values of 1.0 and 1.5 were generated. To isolate the effect of particle AR, all assemblies were prepared with the same relative density of *D*_*r*_ = 42%. Due to differences in particle shape, each assembly exhibited unique minimum and maximum void ratios. To ensure consistency across simulations, samples were standardized by relative density rather than initial void ratio. For spherical particles (AR = 1.0), the minimum and maximum void ratios were ${e}_{\min }=0.58$ and ${e}_{\max }=0.71$, respectively. For elongated particles with AR = 1.5, these values were ${e}_{\min }=0.45$ and ${e}_{\max }=0.72$. Figure 6a shows that reducing the particle AR from 1.5 to 1.0 decreases the maximum deviatoric stress from 135 to 123 kPa. Figure 6b demonstrates that the volumetric response is substantially influenced by particle AR: assemblies with AR = 1.0 exhibit greater volumetric changes at the same level of deviatoric strain compared to those with elongated particles. These results highlight the importance of accounting for particle shape in element-level soil simulations, particularly from a constitutive modeling perspective. The significant influence of particle shape has also been validated by recent DEM simulations and experimental studies (e.g.,^76,77^). The demonstrated control of stiffness, peak strength, and dilatancy by fabric and particle shape echoes granular micromechanics-based modeling successfully extended to advanced composites such as Ultra High Performance Fiber-Reinforced Concrete, reinforcing the generality of micro-informed closures beyond granular soils^78^.

### Deep learning predictions

**Fig. 7:**
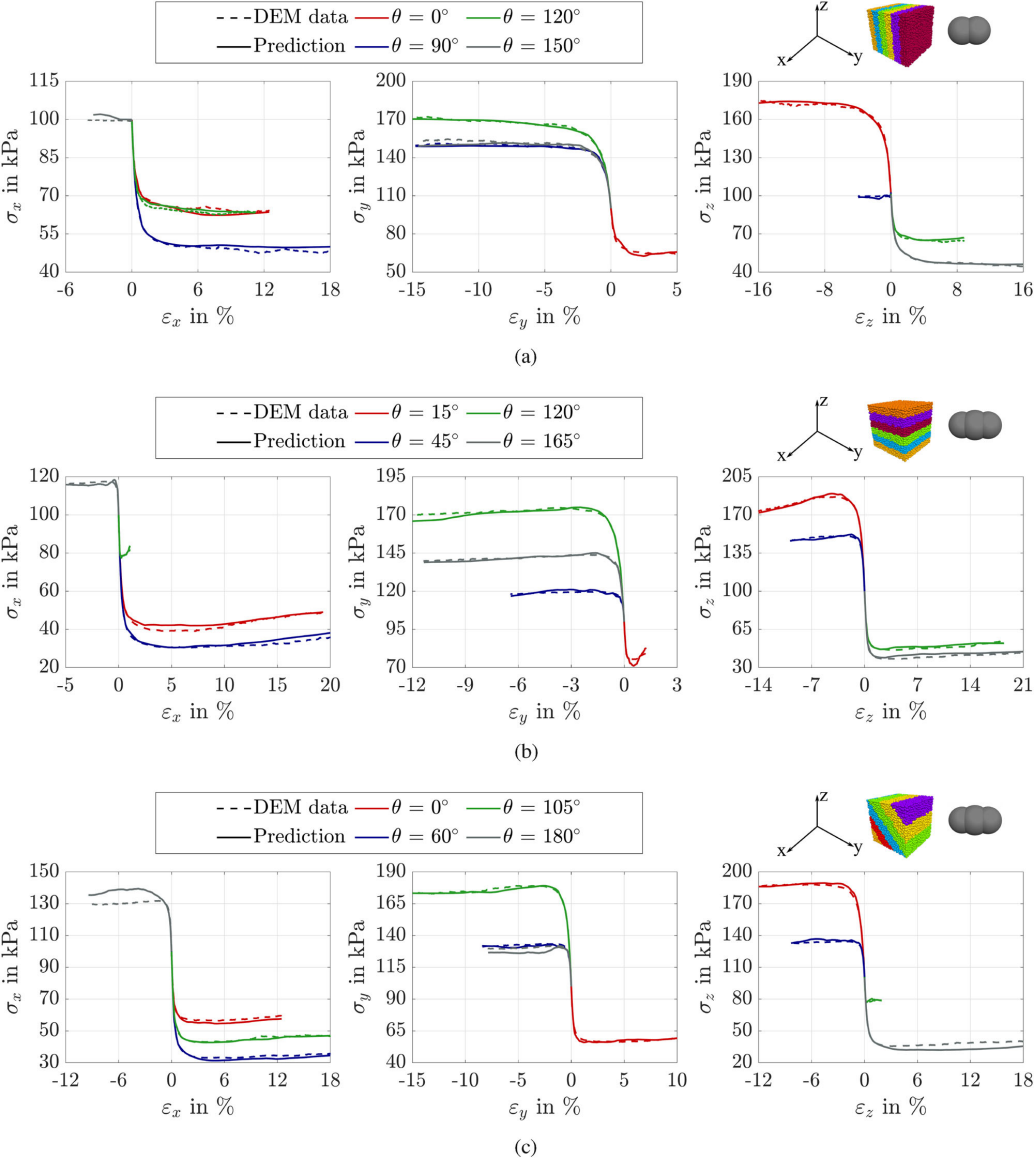
Comparison between proposed model predictions and DEM results. Evolution of stress components *σ*_*x*_, *σ*_*y*_, and *σ*_*z*_ with strain for samples with *p*_0_ = 100 kPa and 0.557≤*e*_0_≤0.589: **a** vertical bedding plane (TR90) with AR = 1.5; **b** horizontal bedding plane (TR0) with AR = 1.8; **c** 45^∘^ bedding plane (TR45) with AR = 1.8

**Fig. 8:**
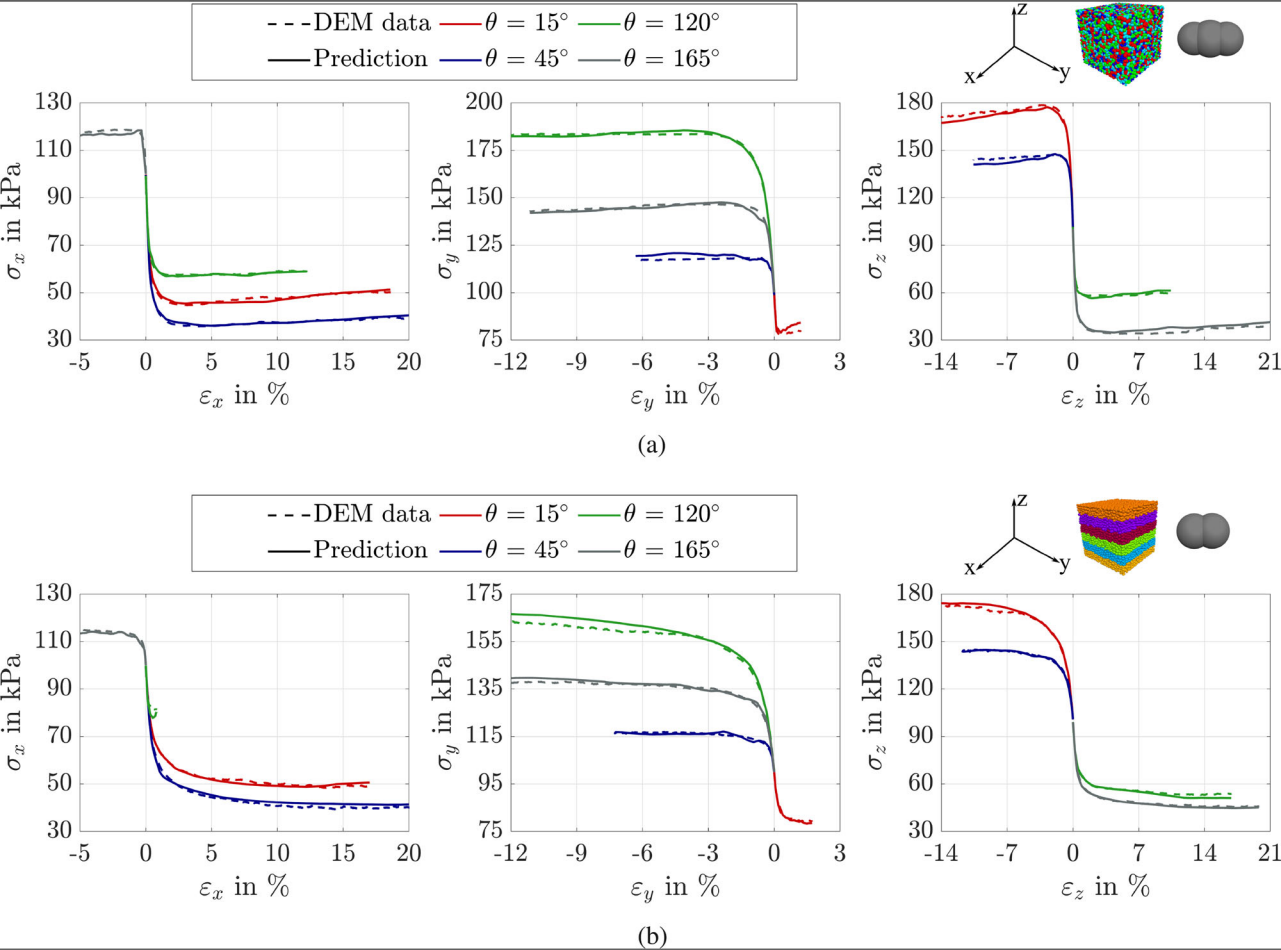
Comparison between proposed model predictions and DEM results. Evolution of stress components *σ*_*x*_, *σ*_*y*_, and *σ*_*z*_ with strain for samples with *p*_0_ = 100 kPa and 0.640≤*e*_0_≤0.689: **a** ISO sample with AR = 1.8; **b** horizontal bedding plane (TR0) with AR = 1.5

Figures 7 and 8 present a comparison between the predictions of the proposed deep-learning model and the DEM simulations for the stress components across assemblies with varying initial stress states subjected to drained true triaxial loading. To ensure the reliability of the findings, the model was trained and validated using a comprehensive dataset derived from 260 DEM data samples. Specifically, 172 data samples were used for training, 13 for validation, and the remaining data samples for testing. For brevity, we present the results for a representative subset of the test data. The model was implemented using the PyTorch framework and trained for 350 epochs. Figure 7a shows that the model accurately predicts the evolution of the stress components in the *x*-, *y*-, and *z*-directions (i.e., *σ*_*x*_, *σ*_*y*_, and *σ*_*z*_), which evolve as functions of the applied strain in the corresponding directions. In addition, the results demonstrate that the deep-learning model successfully predicts direction-dependent stress responses in samples with identical initial mean effective stress (*p*_0_), void ratio (*e*_0_), particle AR, and bedding plane orientation. The predicted stress evolution closely follows the DEM simulations, where the stress response varies significantly with the Lode angle. Importantly, this variation is direction-dependent and does not exhibit a consistent pattern across the x-, y-, and z-directions. For example, in Fig. 7a, the maximum stress in the x-direction for the assembly sheared at a Lode angle of 150° is higher than that for the assembly at 90°. In contrast, the stress in the z-direction follows a different trend, highlighting the anisotropic nature of the stress response under varying Lode angles. The model effectively reproduces the trends observed in the other particulate assemblies shown in Fig. 7b, c. These results indicate that the model successfully captures the anisotropic and path-dependent characteristics of the granular material response. Additionally, the comparison of stress trends in Fig. 7b, c underscores the influence of initial fabric on the directional stress response. Although the samples share identical initial conditions and particle shapes, they follow distinct stress paths, emphasizing the anisotropic effects introduced by fabric orientation. Notably, the deep-learning model successfully captures these fabric-induced variations and closely replicates the trends observed in the corresponding DEM simulations. In this study, we used only the diagonal components of the fabric tensor, as they align with the principal stress directions and were the dominant contributors to the material response.

**Fig. 9:**
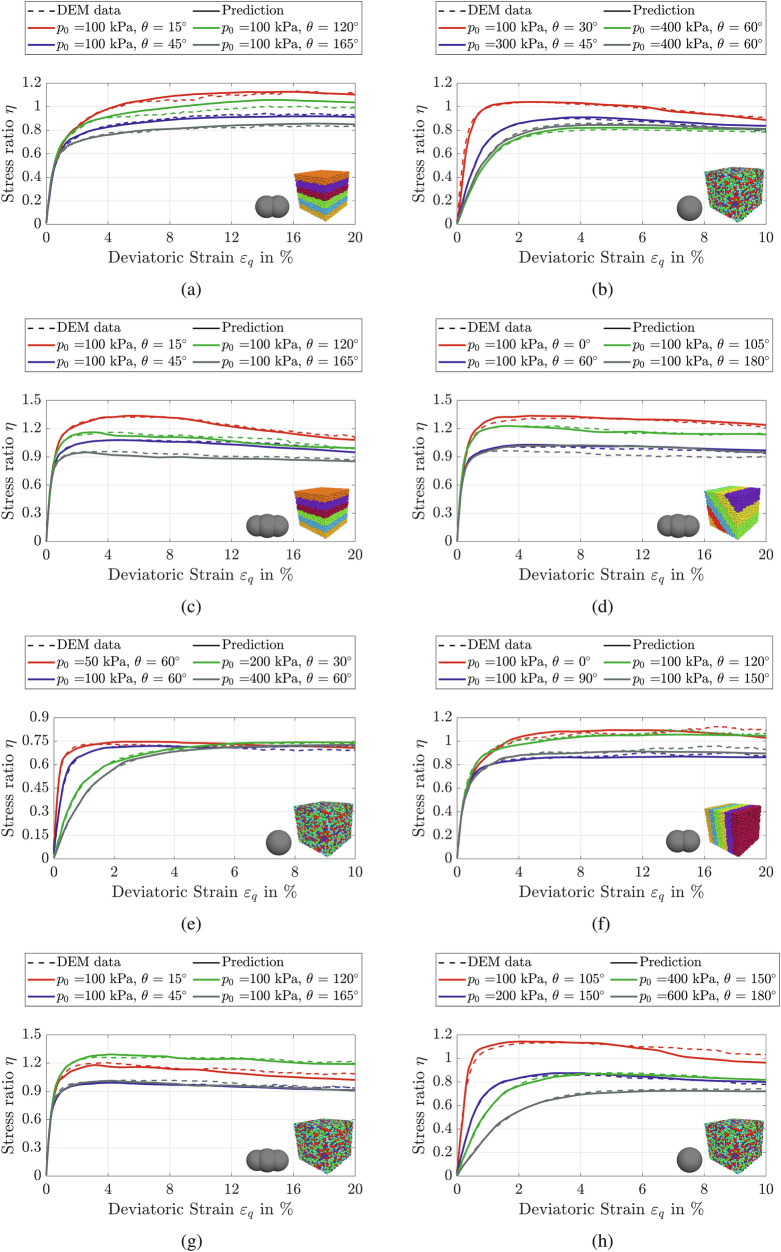
Comparison between the proposed model predictions and DEM results. Evolution of the stress ratio *η* with deviatoric strain *ε*_*q*_ for samples sheared under drained true triaxial conditions with 0.589≤*e*_0_≤0.689: **a** TR0 with AR = 1.5; **b**, **e**, **h** ISO with AR = 1.0; **c** TR0 with AR = 1.8; **d** TR45 with AR=1.8; **f** TR90 with AR = 1.5 and **g** ISO with AR = 1.8.

To facilitate the interpretation of shear behavior, the stress ratio *η* = *q*/*p* is commonly used as a scalar measure of shear strength, as it consolidates the influence of the three principal stresses into a single parameter representing the intensity of shear. Figure 9 presents a comparison between the predictions of the deep-learning model and the corresponding DEM simulations for 32 selected samples, expressed in terms of stress ratio versus deviatoric strain. The results demonstrate that the model accurately captures both the maximum stress ratio and the stress level at the critical state, which is defined as the condition at which soil continues to undergo deformation at constant deviatoric stress and constant void ratio, indicating a stable internal structure. This state is a fundamental concept in critical state soil mechanics and represents the long-term shear strength of the material.

Although the architectural components employed here–feed-forward layers, Leaky-ReLU activation, and standard normalization–are well established, their selection is deliberate and well suited to the static, path-dependent input-output structure of the DEM simulations. This architecture provides a numerically stable and efficient representation of highly nonlinear stress responses without introducing unnecessary complexity from sequential or attention-based models. The use of a weighted smooth-*ℓ*_1_ loss, designed to emphasize small-strain accuracy, together with a PAC-Bayes-based robustness term, prioritizes predictive reliability in the small-strain regime. Overall, the proposed framework enables an end-to-end, data-driven mapping from DEM-derived microstructural descriptors and strain paths to full three-dimensional stress tensors, allowing material anisotropy and Lode-angle dependence to emerge directly from data without imposing constitutive assumptions. It should be noted that the proposed data-driven approach reduces the extensive calibration processes typically required by conventional soil constitutive models, thereby enhancing efficiency in geotechnical engineering practice. Incorporating governing physical principles via a physics-informed loss function is considered a promising direction for future research, with the potential to further enhance the model’s performance. Moreover, microplane and granular micromechanics approaches (e.g.,^79,80^) can also be integrated into our framework to reduce dependence on DEM idealizations. Microplane models may be used to generate complementary stress-strain data for training or validating the deep-learning model, while granular micromechanics provides additional microstructural descriptors (e.g., coordination number, higher-order fabric tensors) that can be incorporated as inputs^81^.

Following validation with element-level tests, the model can be implemented within finite element codes such as ABAQUS as a user-defined material subroutine (UMAT or VUMAT), enabling the simulation of complex boundary value problems in future research. At each integration point and increment, the subroutine should receive the strain increment and the current state variables, including the initial stress tensor, void ratio, and fabric tensor that capture microstructural anisotropy. Using these inputs, the constitutive model predicts the updated stress tensor by integrating the stress-strain response along the given strain path. The current state vector readily accommodates micro-informed elastic bimodulus closures^32,33^ and elasto-plastic-damage or gradient terms grounded in granular energetics^40,41^, enabling systematic extensions within the same UMAT framework. Crucially, the subroutine should also compute the consistent material tangent stiffness matrix, DDSDDE–the derivative of the stress increment with respect to strain increment–which is essential to ensure the quadratic convergence of the global Newton-Raphson iterative solver employed by ABAQUS. The DDSDDE should accurately reflect the coupled evolution of fabric and stress, capturing the anisotropic and path-dependent characteristics of granular soils. By returning both the updated stress and the tangent stiffness matrix at each increment, the subroutine enables the finite element framework to robustly and accurately simulate granular soil behavior under realistic loading and boundary conditions, effectively bridging particle-scale physics with continuum-scale analysis. The present deep-learning model predicts stresses along a prescribed strain history and updates the void ratio directly from the volumetric strain; however, fabric-related internal variables are not updated. As a consequence, while stresses and a strain-consistent tangent stiffness (DDSDDE) can be computed, a fully fabric-coupled tangent suitable for finite element implementation remains a subject for future development.

## Conclusion

This study advances the micro-to-macro understanding of granular material behavior by integrating a comprehensive series of DEM simulations with the development of a novel microstructure-informed deep-learning model. A total of 260 simulations were conducted on granular assemblies with varying initial void ratios, stress states, particle shapes (spherical and elongated with aspect ratios of 1.5 and 1.8), and bedding plane orientations (0°, 45°, and 90°). These simulations systematically capture the influence of microstructural entities on the mechanical response under multidirectional loading conditions with the Lode angle ranging from 0° ≤* θ* ≤ 180°. Building upon this rich dataset, a generalized deep-learning-based constitutive model is proposed that incorporates key micro- and macro-scale descriptors—namely, initial stress state, void ratio, particle AR, and fabric tensor—while continuously tracking the evolving strain path. The deep-learning pipeline comprises map input variables to three continuum-based output stress components, guided by a hybrid data-driven loss function. This model enables accurate prediction of the full tensorial stress response of granular materials, reflecting their inherent anisotropy and complex behavior under true triaxial loading. The simulated loading paths were designed to reflect conditions experienced by geostructures subjected to multidirectional loading due to environmental hazards—such as the foundation of a wind turbine—or even simple slopes, where the principal stresses of the soil elements rotate. Interweaving physics-based DEM modeling with data-driven learning, our approach takes a step forward toward more realistic and reliable simulation-based designs of critical energy transition and infrastructure systems.

## Supplementary information

Supplementary material

## Data Availability

The datasets and materials used and analyzed during the current study are available from the corresponding author upon request.
