# Supplementary material for: Microstructure-informed constitutive modeling of granular media under multidirectional loading: From particle-scale to continuum
Source: Commun Eng. 2026 Apr 30;5:80. doi: 10.1038/s44172-026-00652-1 (PMC13133363; doi:10.1038/s44172-026-00652-1)
Supplement: Supplementary file 1 — Supplementary material [file 44172_2026_652_MOESM1_ESM.pdf]

Supplementary material

A summary of the performed DEM tests is presented in Supplementary Table 1, including the initial state variables, particle shapes, and fabric tensor components of the assemblies. The samples were sheared under drained true triaxial conditions with Lode angle values ranging from 0° to 180°. It is worth noting that the components of (**F**) function as a measurable proxy of fabric for constitutive state definition and calibration.

Supplementary Table 1: Illustration of sample deposition types, particle shapes, initial mean effective stress p_0, void ratios e_0, and fabric tensor components $\mathbf{F}$ for samples subjected to drained true triaxial tests with Lode angle ranging from 0° to 180°

Illustration of sample deposition types	$e_0 \approx$	p_0 (kPa) $\approx$	$\mathrm{F_x} \approx$	$\mathrm{F_y} \approx$	$\mathrm{F_z} \approx$	Particle shape
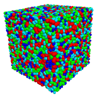<br>ISO	0.72<br>0.68<br>0.59<br>0.67<br>0.65<br>0.67<br>0.59<br>0.63<br>0.60<br>0.64<br>0.59	50.0<br>50.0<br>100.0<br>100.0<br>200.0<br>200.0<br>300.0<br>300.0<br>400.0<br>400.0<br>600.0	0.3340<br>0.3332<br>0.3336<br>0.3340<br>0.3325<br>0.3336<br>0.3333<br>0.3326<br>0.3324<br>0.3347<br>0.3350	0.3326<br>0.3358<br>0.3327<br>0.3332<br>0.3352<br>0.3330<br>0.3332<br>0.3345<br>0.3350<br>0.3318<br>0.3334	0.3334<br>0.3317<br>0.3337<br>0.3328<br>0.3323<br>0.3334<br>0.3335<br>0.3329<br>0.3326<br>0.3336<br>0.3317	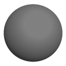
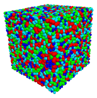<br>ISO	0.59<br>0.54<br>0.70<br>0.68<br>0.63	50.0<br>100.0<br>100.0<br>100.0<br>100.0	0.3419<br>0.3455<br>0.3472<br>0.3440<br>0.3634	0.3277<br>0.3266<br>0.3243<br>0.3279<br>0.3083	0.3304<br>0.3279<br>0.3285<br>0.3281<br>0.3284	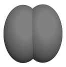
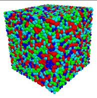<br>ISO	0.71<br>0.67<br>0.64<br>0.59<br>0.53	50.0<br>50.0<br>100.0<br>100.0<br>100.0	0.3343<br>0.3323<br>0.3492<br>0.3475<br>0.3450	0.3333<br>0.3359<br>0.3223<br>0.3262<br>0.3250	0.3323<br>0.3318<br>0.3284<br>0.3262<br>0.3310	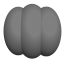
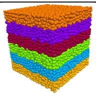<br>TR0	0.69<br>0.54<br>0.69	100.0<br>100.0<br>200.0	0.3149<br>0.3426<br>0.3117	0.3096<br>0.3305<br>0.3106	0.3755<br>0.3269<br>0.3777	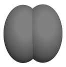
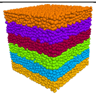<br>TR0	0.59	100.0	0.3067	0.3012	0.3912	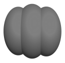
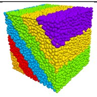<br>TR45	0.69<br>0.69	100.0<br>200.0	0.3291<br>0.3358	0.3143<br>0.3078	0.3566<br>0.3565	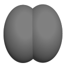
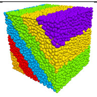<br>TR45	0.59	100.0	0.3067	0.3012	0.3912	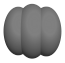
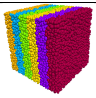<br>TR90	0.69<br>0.64<br>0.69	100.0<br>100.0<br>200.0	0.3682<br>0.3634<br>0.3671	0.3042<br>0.3083<br>0.3029	0.3276<br>0.3284<br>0.3300	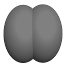
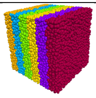<br>TR90	0.59	100.0	0.3676	0.3141	0.3183	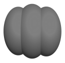
